# Effect of Growth Hormone on Branched‐Chain Amino Acids Catabolism in Males With Hypopituitarism

**DOI:** 10.1111/jcmm.70451

**Published:** 2025-03-03

**Authors:** Yuwen Zhang, Zhiqiu Ye, Enfei Xiang, Peizhan Chen, Xuqian Fang

**Affiliations:** ^1^ Department of Endocrine and Metabolic Diseases Shanghai Institute of Endocrine and Metabolic Diseases, Ruijin Hospital, Shanghai Jiao Tong University School of Medicine Shanghai China; ^2^ Shanghai National Clinical Research Center for Metabolic Diseases, Key Laboratory for Endocrine and Metabolic Diseases of the National Health Commission of the PR China Shanghai National Center for Translational Medicine, Ruijin Hospital, Shanghai Jiao Tong University School of Medicine Shanghai China; ^3^ Department of Pathology Ruijin Hospital, Shanghai Jiao Tong University School of Medicine Shanghai China; ^4^ Clinical Research Center Ruijin Hospital, Shanghai Jiao Tong University School of Medicine Shanghai China

**Keywords:** 4D label‐free quantitative phosphoproteomics, branched‐chain amino acids degradation, growth hormone deficiency, hypopituitarism

## Abstract

To investigate the impact of growth hormone (GH) on branched‐chain amino acids (BCAAs) catabolism in males with hypopituitarism, we measured the concentration of amino acids in 133 males with hypopituitarism and 90 age‐matched healthy controls using untargeted metabolome. A rat model of hypopituitarism was established through hypophysectomy, followed by recombinant human GH (rhGH) intervention. Targeted metabolomics and label‐free quantitative phosphoproteomics were utilised to assess amino acid levels in rats and explore the mechanisms of GH's effect on BCAA catabolism. Hypopituitarism exhibited elevated concentrations of BCAAs, which correlated positively with triglyceride, fasting insulin and HOMA‐IR. The BCAAs were significantly elevated following hypophysectomy and were substantially reduced upon rhGH intervention. Phosphorylation proteomics analysis in liver tissues revealed that differentially expressed phosphoproteins (DEPPs) after GH treatment were predominantly involved in ‘RNA metabolic process’, ‘Diseases of signal transduction by growth factor receptors’ and ‘BCAAs degradation’. Notably, 12 proteins in the BCAA degradation pathway showed altered phosphorylation without whole protein changes. Importantly, the expression or phosphorylation modification of BCKDH, BCATs and MuRF1 were restored through rhGH intervention. Hypopituitarism exhibits elevated levels of circulating BCAAs. The increased circulating BCAAs in hypopituitarism may result from enhanced MuRF1‐mediated muscle proteolysis, which greatly exceeds the BCAA degradation capacity. This study provides valuable insights into the effects of GH on BCAA catabolism at the scale of the proteomics level.

## 1 Introduction

Hypopituitarism is characterised by the partial or complete loss of anterior pituitary hormones, including GH, luteinising hormone (LH), follicle‐stimulating hormone (FSH), adrenocorticotropic hormone (ACTH) and thyrotropin (TSH) [1]. Due to GH deficiency (GHD), hypopituitarism patients exhibit increased visceral adiposity, IR, dyslipidaemia and hyperglycaemia, increasing the incidence and mortality rate of cardiovascular diseases [2, 3].

Skeletal muscle, a target organ of GH, experiences atrophy and metabolic disorders in the absence of adequate GH levels [4]. In adults with GHD, lean body mass (LBM) and muscle mass are reduced due to disrupted protein metabolism [5]. GH replacement therapy in GHD stabilises protein metabolism by favouring protein synthesis pathways over amino acid oxidation [6, 7]. Muscle atrophy is associated with increased expression of muscle atrophy F‐box protein (MAFbx) and muscle‐specific RING finger 1 (MuRF1), which induce ubiquitination and proteasome‐mediated degradation of target proteins, resulting in rapid muscle mass loss [8]. Russell‐Jones et al. [9] observed that GH supplementation in growth hormone deficiency (GHD) subjects led to an increase in protein synthesis and a reduction in protein oxidation. GH replacement therapy was found to restore protein stabilisation by favouring amino acid utilisation in protein synthesis pathways, thereby ameliorating muscle mass loss. Despite these positive effects on protein metabolism, the specific role of GH in modulating the ubiquitin‐proteasome system during this process remains unknown.

Branched‐chain amino acids (BCAAs), namely leucine, isoleucine and valine, are indispensable amino acids that mammals cannot synthesise de novo. Consequently, they must be acquired through dietary intake, as a subset of enzymes essential for their biosynthesis is lacking in human and other mammalian tissues. Amino acids derived from dietary proteins are transported through the circulation to skeletal muscle, where they play a pivotal role in synthesising essential proteins [10]. Elevated concentrations of BCAAs have been implicated in the pathogenesis of IR, type 2 diabetes (T2D) and various cardiometabolic diseases [11]. In parallel with the clinical features observed in obesity and T2D, hypopituitarism presents with characteristics such as central obesity, IR and an increased susceptibility to cardiovascular diseases. The exploration of the intricate interplay between BCAAs and metabolic disturbances in hypopituitarism will provide valuable insights into potential mechanisms underlying these clinical features.

Several studies have proposed that the elevated circulating BCAAs observed in patients with IR may result from dysregulated BCAA oxidation pathways in adipose and hepatic tissues, primarily influenced by impaired functions of BCAT and BCKDH [12]. In murine models of obesity and IR, levels of valine and leucine/isoleucine have been reported to increase by 20% and 14%, respectively. This rise in BCAAs has been linked to the downregulation of multiple enzymes in the oxidation pathway [13, 14]. This transcriptional downregulation of BCAA oxidation enzymes has also been observed in human participants with obesity, which can be reversed by weight loss surgery and accompanied by a decrease in circulating BCAA levels [15]. In cases of obesity and IR, minimal changes are observed in BCKDH abundance in the liver. Instead, BCKDH activity is primarily impaired by the induction of BDK and repression of PPM1K, leading to hyperphosphorylation of BCKDH and subsequent inhibition of its enzymatic activities [16]. Moreover, the transplantation of normal adipose tissue into mice lacking BCAT2 has been demonstrated to reduce circulating BCAA levels by 30%–50% [14, 17]. Collectively, these findings suggest an increase in circulating BCAAs in obesity and diabetes, potentially attributed to impaired BCAA oxidation pathways resulting from decreased expression or altered phosphorylation of BCAA oxidation enzymes.

To investigate the impact of GH on BCAAs catabolism in males with hypopituitarism, the current study conducted a case–control investigation involving 133 individuals with hypopituitarism and 90 paired controls. Furthermore, we also established animal models of hypopituitarism (hypophysectomized rats). This study provides an in‐depth understanding of the mechanisms underlying GH's effect on BCAAs catabolism in hypopituitarism.

## 2 Materials and Methods

### 2.1 Participant Recruitment

Patients and healthy controls were recruited at Ruijin Hospital in Shanghai, China, between January 2016 and December 2018. The recruitment process for the current study adhered to the same protocol as a previously published study [18], including congenital hypopituitarism and acquired hypopituitarism, with further exclusion criteria of patients with normal GH levels. The study protocol received approval from the Board of Medical Ethics at Ruijin Hospital, Shanghai Jiao Tong University School of Medicine, China. Ethical considerations and patient confidentiality were maintained throughout the recruitment process and the entire duration of the study.

### 2.2 Hormone Replacement Plan of Patients With Hypopituitarism

Physiologic dosages of glucocorticoids and/or thyroid hormone were administered after diagnosis (median diagnosis age 16.50 year). A subset of these patients had undergone GH replacement during their childhood, subsequently discontinuing the treatment for a minimum of 24 months. Gonadotropin treatment was administered to all patients with LH/FSH deficiency for at least 24 months.

### 2.3 Measurement of Biochemical Markers in Patients With Hypopituitarism

Serum total cholesterol, high‐density lipoprotein cholesterol, low‐density lipoprotein cholesterol and triglycerides were measured by the automated enzymatic method on an autoanalyser (Beckman Coulter, California, USA). Plasma glucose was measured by the glucose oxidase method with the same autoanalyser. Serum insulin was measured by a commercially available RIA kit (Diagnostic Systems Laboratories, Minnesota, USA). IR was quantified using the homeostasis model assessment of insulin resistance (HOMA‐IR) index (HOMA‐IR = insulin [μU/mL] × glucose [mmol/L]/22.5).

### 2.4 Amino Acids Quantification in Patients With Hypopituitarism Through Untargeted Metabolomics

Metabolic profiling of serum samples was conducted on an Agilent 1290 Infinity LC system (Agilent Technologies, California, USA) coupled with an AB SCIEX Triple TOF6600 system (AB SCIEX, California, USA) to measure the amino acid levels. Chromatographic separation was performed on an ACQUITY HSS T3 1.8 μm column. Variable importance in projection (VIP) values for each variable in the orthogonal partial least squares‐discriminant analysis (OPLS‐DA) model were calculated to identify metabolites contributing to classification. Variables with *p*‐values < 0.05 and VIP values > 1 were considered statistically significant. Our method allows for a relative quantitative analysis of the AA profile through untargeted metabolomics based on peak areas. The levels of AAs were recorded based on the peak area.

### 2.5 Hypophysectomized Rat Models and GH Intervention

Male Sprague–Dawley (SD) rats aged 3–4 weeks (weighing 70–80 g) were equally randomised into three groups. The hypophysectomy was performed according to the following procedure. A 1.5 to 2.5 cm incision was made along the midline of the neck of the rat, starting from the lower jaw nipple downward. The incision was made bluntly, following the direction of muscle fibres, to separate the subcutaneous tissue and salivary glands, exposing the trachea. The anterior neck fascia was opened on the left or right side of the midline, and the salivary glands were pulled aside. A syringe needle was inserted between the 3rd and 4th tracheal cartilage rings, and after the needle was withdrawn, a PE50 or PE90 tube was inserted along the needle track. The muscle tissue and trachea were pulled apart on both sides to fully expose the base of the skull. The meninges and adhering tissues on the base of the skull were scraped off to expose the sphenoparietal suture. A drill was used to create a hole in the base of the skull, allowing access to the cranial cavity. After removing the membrane covering the surface of the pituitary gland, a suction tube connected to a negative pressure pump (at 320–500 mmHg) was used to aspirate the pituitary gland. After haemostasis was achieved with cotton swabs and gauze in the operative area, the surgical retractor was removed. After the animal regained breathing, the endotracheal tube was removed. The sternocleidomastoid muscle was retracted to fully cover the tracheotomy incision, and the skin incision was sutured. Before the rat regained consciousness, attention should be paid to secretions in the oral cavity or trachea to prevent asphyxiation. After regaining consciousness, clean water should be provided and subcutaneous injection of 10–15 mL of Ringer's solution or glucose saline solution may be considered for rats with significant blood loss during surgery. Because the energy metabolism level of the hypophysectomized rat is lower, attention should be paid to insulation.

The success criterion for the model was defined as a postoperative body weight gain of less than 10% after 2 weeks. The rats in the control group were subjected to a sham operation, which involved the administration of general anaesthesia, the cutting and subsequent suturing of the skin on the neck.

After 2 weeks' recovery, one of the hypophysectomized groups was provided rhGH (0.1 mg/kg/day) (Genlei, Changchun, China) daily at a relatively fixed time of 11:00–12:00 am with subcutaneous injection, including weekends for 2 weeks [ 19]. The other groups were injected with 100 μL physiological saline. The dose and duration of rhGH replacement were determined based on the physiological replacement doses of GH in adult GHD. Body weight and fasting blood samples were collected before and 2 weeks after the operation, and 2 weeks after rhGH intervention. At the end of the study, the overnight‐fasted rats were euthanized. The livers and extensor digitorum longus muscles were excised and frozen immediately in liquid nitrogen. Blood samples were collected, and plasma was obtained by centrifugation (2200× *g*, 4°C) and stored at −80°C.

### 2.6 Amino Acids Quantification in the Serum of Rats by Targeted Metabolomics

The concentrations of serum amino acids were determined by high‐performance liquid chromatography/mass spectrometry. In brief, 80 μL of an ethylene diamine tetraacetic acid sample was deproteinized with 1 mL of methanol and subsequently purified through ion exchange columns. Statistical significance was determined for metabolites based on *p*‐values < 0.05 and VIP values > 1. A heatmap visualising the differential expression of these metabolites was constructed across the time points. The LC–MS analysis was conducted by Applied Protein Technology Co. Ltd. (APTBIO, Shanghai, China).

### 2.7 Four‐Dimensional (4D)‐Label Free Phosphorylation Proteomics

The protein concentration was quantified using the BCA Protein Assay Kit (Beyotime Biotechnology, Shanghai, China). For filter‐aided sample preparation, 200 μg of protein was mixed with 30 μL of SDT buffer. The phosphopeptides were enriched using the High‐Select Fe‐NTA Phosphopeptides Enrichment Kit (Thermo Scientific, Massachusetts, USA). LC–MS/MS analysis was performed on a timsTOF Pro mass spectrometer coupled to Nanoelute (Bruker, Massachusetts, USA) for 60 min. 4D label‐free phosphorylation proteomics was conducted at Applied Protein Technology Co. Ltd. The differentially expressed phosphoproteins (DEPPs) were identified with standards of *p*‐values < 0.05 and VIP values > 1. The gene set enrichment analysis for differentially expressed phosphoproteins was performed using the Kyoto Encyclopedia of Genes and Genomes (KEGG) database, which was automatically generated by Metascape (https://metascape.org).

### 2.8 Western Blotting

Tissue homogenates were lysed in RIPA lysis buffer supplemented with protease inhibitor cocktail (APExBio, Houston, USA). Approximately 50–70 μg protein was separated by 6%–12% SDS‐PAGE, transferred to PVDF membranes (Bio‐Rad, Hercules, USA), and probed with primary antibodies. The antibodies of BCAT1 D6D4K (88785), BCAT2 D8K3O (79764), BCKDH‐E1α E4T3D (90198) and Phospho‐BCKDH‐E1α (Ser293) E2V6B (40368) all sourced from Cell Signalling Technology (CST, Massachusetts, USA), while MURF1 ab183094 was acquired through Abcam (Abcam, Massachusetts, USA).

### 2.9 Calculation of Muscle Cross‐Sectional Area (CSA)

The CSA was calculated assuming the cross‐section to be approximately elliptical. The formula for the area of an ellipse was used, *A* = π × *L*/2 × *W*/2, where *A* represents area, *L* is the major axis, *W* is the minor axis and *π* is approximately 3.14. The CSA was calculated for 30 cells that were approximately elliptical in shape, and the mean value was determined. One‐way ANOVA was employed to analyse the muscle CSA among three groups of animals.

### 2.10 Assessment of Cell Density in Skeletal Muscle

To evaluate cell density in skeletal muscle, regions of 0.050 mm^2^ were selected from HE‐stained images. The number of skeletal muscle cells within these regions was counted. For cells located at the edges of the selected areas, only those with more than half of their volume within the region were included in the count. Four random regions with the same areas were selected, and the mean number of cells was calculated from these counts.

### 2.11 Statistical Analysis

The Kolmogorov–Smirnov statistical test was performed to assess data normality. Continuous variables were presented as the mean ± SD for normally distributed variables or medians (interquartile ranges) for the skewed variables. For multiple metabolites comparisons, including 13 amino acids, in metabolome analysis, ‘*q*‐value’ was used instead of the *p*‐value, which has been adjusted using the False Discovery Rate (FDR) method. For comparison of multiple groups, one‐way analysis of variance (ANOVA) was used, followed by Tukey's honest significant difference post hoc test. The correlations between amino acids and metabolism parameters were assessed using Pearson's coefficient. The diagnostic abilities of valine and leucine in hypopituitarism patients were assessed with a receiver operating characteristic (ROC) curve and the area under the ROC curve (AUC). Analyses were performed with Graphpad Prism 8.0 (Graphpad, California, USA) or R software. The significance tests were two‐tailed, and statistical significance was set at *p* < 0.05.

## 3 Results

### 3.1 Physical and Biological Characteristics of Hypopituitarism

There were no significant differences observed in age, height, weight and BMI between individuals with hypopituitarism and their age, male‐matched controls. However, elevated levels of fasting triglycerides, total cholesterol, glucose, insulin and HOMA‐IR were noted in individuals with hypopituitarism compared to the control group (Table 1).

**TABLE 1:** Baseline characteristics of hypopituitarism and healthy participants.

Characteristics	Hypopituitarism	Healthy controls	*p*‐value*
	(*n* = 133)	(*n* = 90)	
Baseline parameters			
Gender (male/female)	133/0	90/0	/
Age (year)	24.50 ± 5.98	24.13 ± 3.28	0.147
Congenital/acquired	82/51	/	/
Height (cm)	166.20 ± 8.36	167.70 ± 5.45	0.212
Weight (kg)	65.38 ± 12.98	66.79 ± 4.70	0.323
BMI (kg/m^2^)	24.02 ± 4.08	23.79 ± 1.96	0.618
GH (ng/mL)	0.045 (0.015–0.084)	/	/
IGF‐1 (ng/mL)	46.0 (28.0–79.0)	/	/
Pituitary hormone deficiency			/
GH deficiency	100% (133/133)	/	/
LH/FSH deficiency	97.74% (130/133)	/	/
TSH deficiency	93.98% (125/133)	/	/
ACTH deficiency	92.48% (123/133)	/	/
Hormone treatments			
GH	0% (0/0)	/	/
Gonadotropin	97.74% (130/133)	/	/
Thyroid hormone	93.98% (125/133)	/	/
Glucocorticoid	92.48% (123/133)	/	/
Glycolipid metabolism (Median, IQR)			
Triglycerides (mmol/L)	1.61 (0.99–2.63)	0.96 (0.64–1.33)	< 0.0001
Total cholesterol (mmol/L)	4.88 (4.19–5.67)	4.53 (4.02–5.11)	0.004
Fasting glucose (mmol/L)	4.75 (4.44–5.13)	4.40 (4.20–4.70)	< 0.0001
Insulin fasting (μU/mL)	14.57 (10.38–20.40)	10.93 (7.38–13.63)	< 0.0001
HOMA‐IR	2.97 (2.14–4.47)	2.04 (1.49–2.83)	< 0.0001

*Note:* Variables were presented as mean ± SD for normally distributed data, or as median (IQR) for non‐normally distributed data.

Abbreviations: ACTH, adrenocorticotropic hormone; BMI, body mass index; FSH, follicle‐stimulating hormone; GH, growth hormone; HOMA‐IR, homeostasis model assessment for IR; LH, luteinizing hormone; TSH, thyroid‐stimulating hormone.

* The *p*‐values of Student's *t*‐test for basic information, *p*‐values of Mann–Whitney test for glycolipid metabolism.

### 3.2 Increased Circulating BCAAs in Hypopituitarism

**FIGURE 1:**
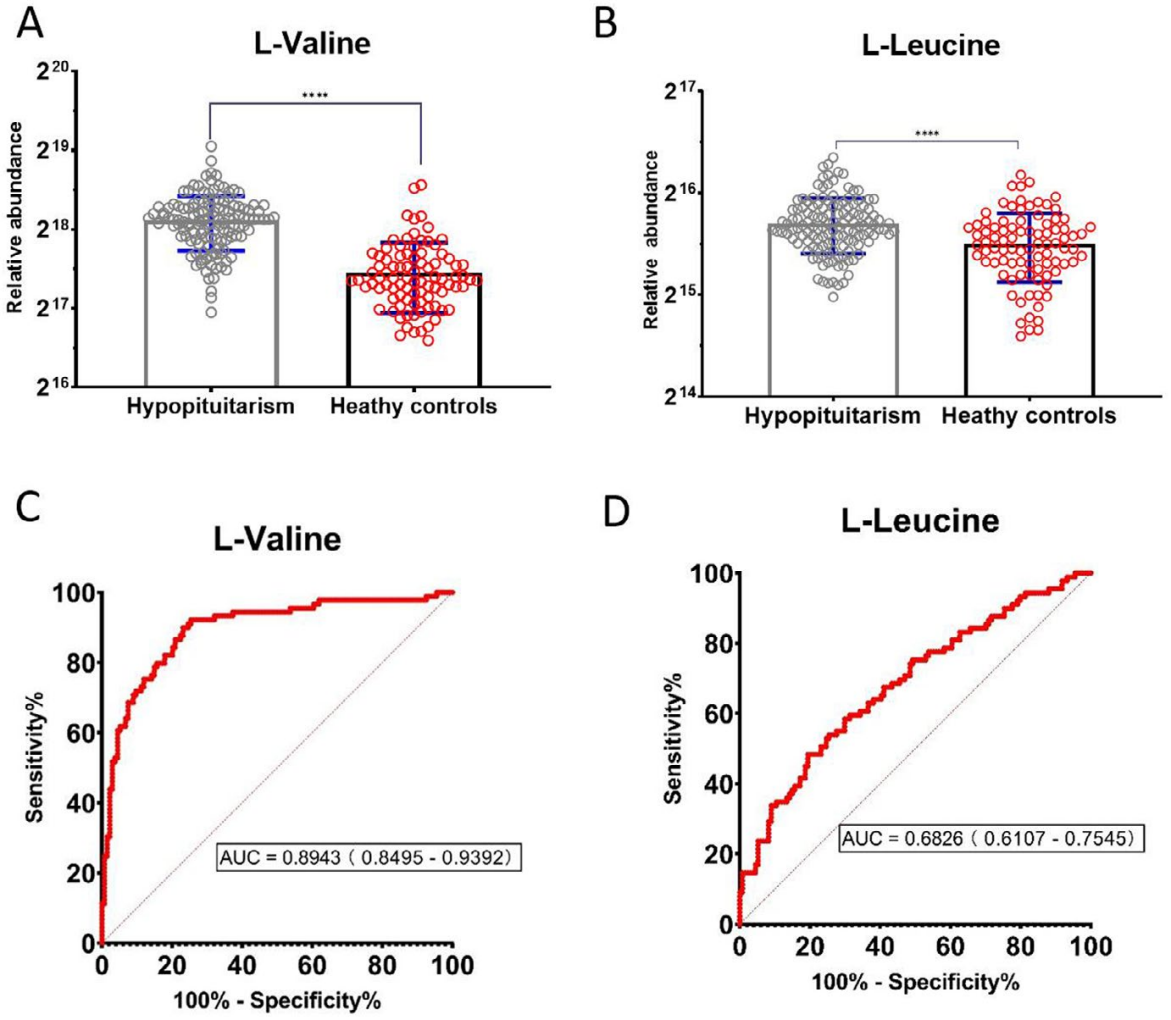
Comparison of fasting plasma levels of L‐valine (A) and L‐leucine (B) in hypopituitarism, compared to age‐paired healthy controls. ****p < 0.0001 for unpaired t test. The ROC tests of L‐valine (C) and L‐leucine (D) for hypopituitarism patients' diagnosis.

In total, thirteen amino acids and metabolites displayed differential abundances in patients with hypopituitarism. Notably, higher levels of alanine, arginine, glutamate, histidine, leucine, phenylalanine, proline, pyroglutamic acid, tryptophan and valine were observed, while glutamine, lysine and norleucine levels were lower in hypopituitarism compared to those in healthy controls (Table 2). Significantly higher levels of valine and leucine were observed in hypopituitarism, with a 1.15‐fold increase (*p* < 0.001) for leucine and a 1.57‐fold increase (*p* < 0.001) for valine. Valine displayed promising potential as a diagnostic biomarker, with an area under the curve (AUC) of 0.8943 (95% CI = 0.8495–0.9392) (Figure 1).

**TABLE 2:** Comparison of amino acid and metabolite levels in the serum of hypopituitarism and healthy participants.

Metabolites	Hypopituitarism	Healthy controls	Hypopituitarism vs. Healthy controls		
	Mean^a^	Mean^a^	VIP	FC	*q*‐value
Alanine	29885.86	2966.88	1.57	7.97	< 0.000001
Arginine	1,440,580	1,060,962	4.30	1.29	0.000001
Glutamate	70889.50	37999.24	2.12	1.96	0.000002
Glutamine	7931.799	22769.86	1.13	0.39	< 0.000001
Histidine	83363.29	55075.55	1.52	1.39	< 0.000001
Leucine	52197.40	46466.59	1.01	1.15	< 0.000001
Lysine	59787.60	222384.80	4.15	0.34	< 0.000001
Norleucine	6283.68	21685.95	1.31	0.33	< 0.000001
Phenylalanine	292730.30	197296.70	3.60	1.43	< 0.000001
Proline	134412.20	93862.68	1.63	1.47	< 0.000001
Pyroglutamic acid	491450.70	352649.30	4.02	1.28	< 0.000001
Tryptophan	115055.40	63438.76	2.18	1.52	0.000001
Valine	285274.90	168461.80	4.04	1.57	0.000002

*Note:* VIP is a statistical measure used in multivariate statistical analysis, particularly within the context of Partial Least Squares (PLS) and Orthogonal Projections to Latent Structures (OPLS) models. VIP is employed to assess the significance and importance of predictor variables on the model's response.

Abbreviations: FC, fold‐change; VIP, variable important in projection.

^a^ The levels of AAs were recorded based on the peak area.

### 3.3 Increased Circulating BCAAs Significantly Correlated With IR in Hypopituitarism

**FIGURE 2:**
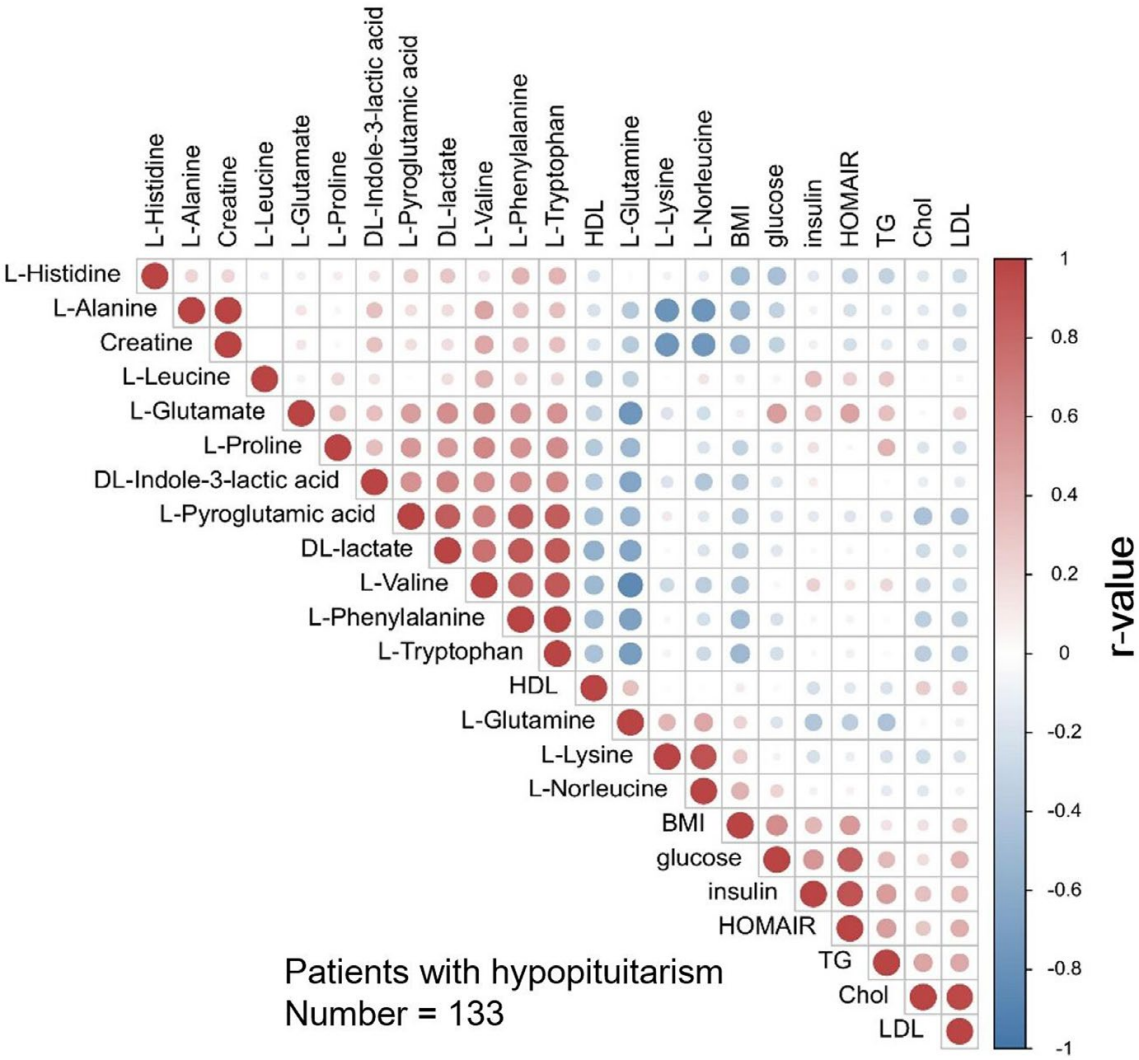
Pearson correlation between amino acids and biomarkers in carbohydrate, lipid metabolism in hypopituitarism. The differential expression levels of amino acids and biomarkers in carbohydrate, lipid metabolism were processed using *Z*‐score data normalisation. Different colours were used to indicate the correlation results: Red represented positive correlation, blue represented negative correlation, and white indicated non‐significant correlation. The colour gradient was proportional to the absolute value of the correlation coefficient, with darker colours indicating stronger correlation. The size of the circles was associated with the significance of the correlation: The smaller the *p*‐value, the larger the circle size.

Among the amino acids, valine, leucine and glutamate exhibited positive correlations with biomarkers related to lipid and carbohydrate metabolism (Figure 2). Specifically, the concentration of valine was positively correlated with triglycerides, insulin and HOMA‐IR (*r* = 0.201, *p* < 0.05; *r* = 0.278, *p* < 0.01; *r* = 0.265, *p* < 0.01) and the concentration of leucine was also positively correlated with these biomarkers. Moreover, a positive correlation was observed between glutamate and triglycerides, LDL, glucose, insulin and HOMA‐IR. In the healthy control group, no significant correlation was observed between valine, leucine or glutamate and HOMA‐IR.

### 3.4 Hepatic Steatosis, CSA and IR in Hypophysectomized Rats

**FIGURE 3:**
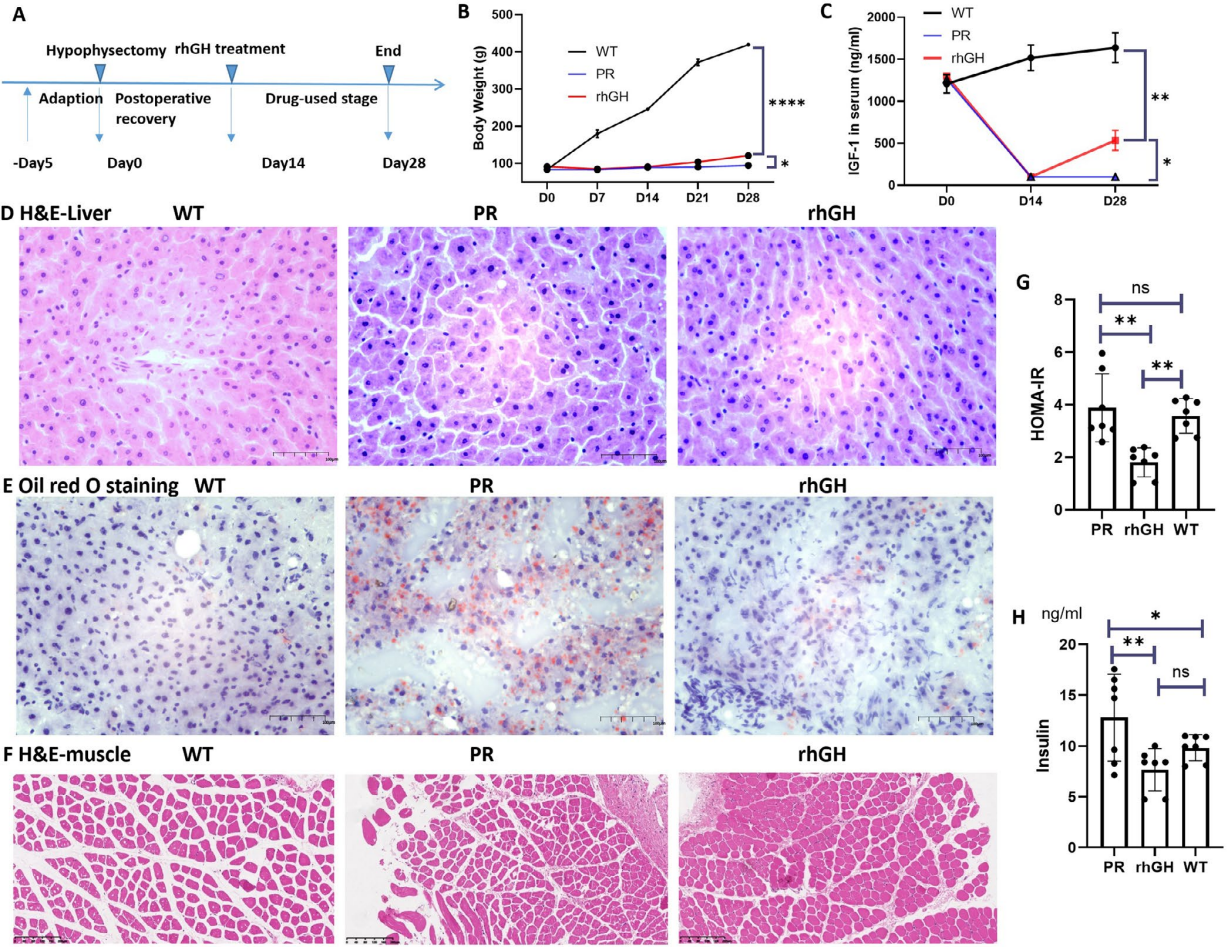
Morphologic and biochemical changes in hypophysectomized rat model. The study design of hypophysectomized rats and rhGH intervention (A); Effects of GH on body weight (B), IGF‐1 expression (C), analysed by two way ANOVA; Pathological HE staining of liver tissues (D), Oil Red O staining of liver tissues (E), HE staining of muscle tissues (F), fasting glucose (G) and fasting insulin (H) in control group (WT), hypophysectomized rats (PR) and rhGH intervention group (rhGH). ****p < 0.0001, ***p < 0.001, **p < 0.01, *p < 0.05.

Hypophysectomized rats were utilised as the animal model for investigating the effects of rhGH intervention (Figure 3A). Following hypophysectomy, the rats exhibited a noticeable decrease in appetite and experienced a body weight gain of less than 10% within 2 weeks (Table S1). However, the administration of rhGH during the subsequent two‐week period resulted in significant weight recovery (Figure 3B). The successful extraction of the pituitary gland was also confirmed by serum IGF‐1 levels, which decreased by over 80% in hypophysectomized rats (PR group) and significantly increased in the rhGH group (Figure 3C). Liver tissue analysis revealed hepatic steatosis in the PR group, which was partly restored after rhGH intervention, as evidenced by Oil Red O staining in liver tissue (Figure 3D,E). Morphologically, the muscle cells in the WT group were relatively loosely distributed, while those in the PR and rhGH groups were closely packed, as depicted in Figure 3F. However, the weight and volume of thigh/body significantly decreased in the PR group, as evidence of enhanced proteolysis (Figure S1). The mean CSA of muscle cells for the WT group was 1630 ± 319.6 μm^2^, for the PR group was 1548 ± 431.4 μm^2^, and for the rhGH group was 1266 ± 266.1 μm^2^. The WT group had the comparatively largest CSA, while the rhGH group had the comparatively smaller CSA area (Table S3). The curve of CSA frequency distribution was shown in Figure S2. Morphologically, the muscle cells in the WT group were relatively loosely distributed, while those in the PR and rhGH groups were closely packed. In an area of 0.050 mm^2^, the cell count for the WT group was 19.75 ± 0.96, for the PR group was 26.25 ± 2.63, and for the rhGH group was 31.60 ± 2.65 (Figure S3–S5). There were significant differences among the three groups. The cell density in the PR and rhGH groups was notably increased. HOMA‐IR was relatively higher in the PR group compared to the rhGH group (Figure 3G). The fasting insulin concentration was paradoxically highest in the PR group, a condition that was mitigated by rhGH replacement (Figure 3H).

### 3.5 Increased BCAAs in Hypophysectomized Rats

**FIGURE 4:**
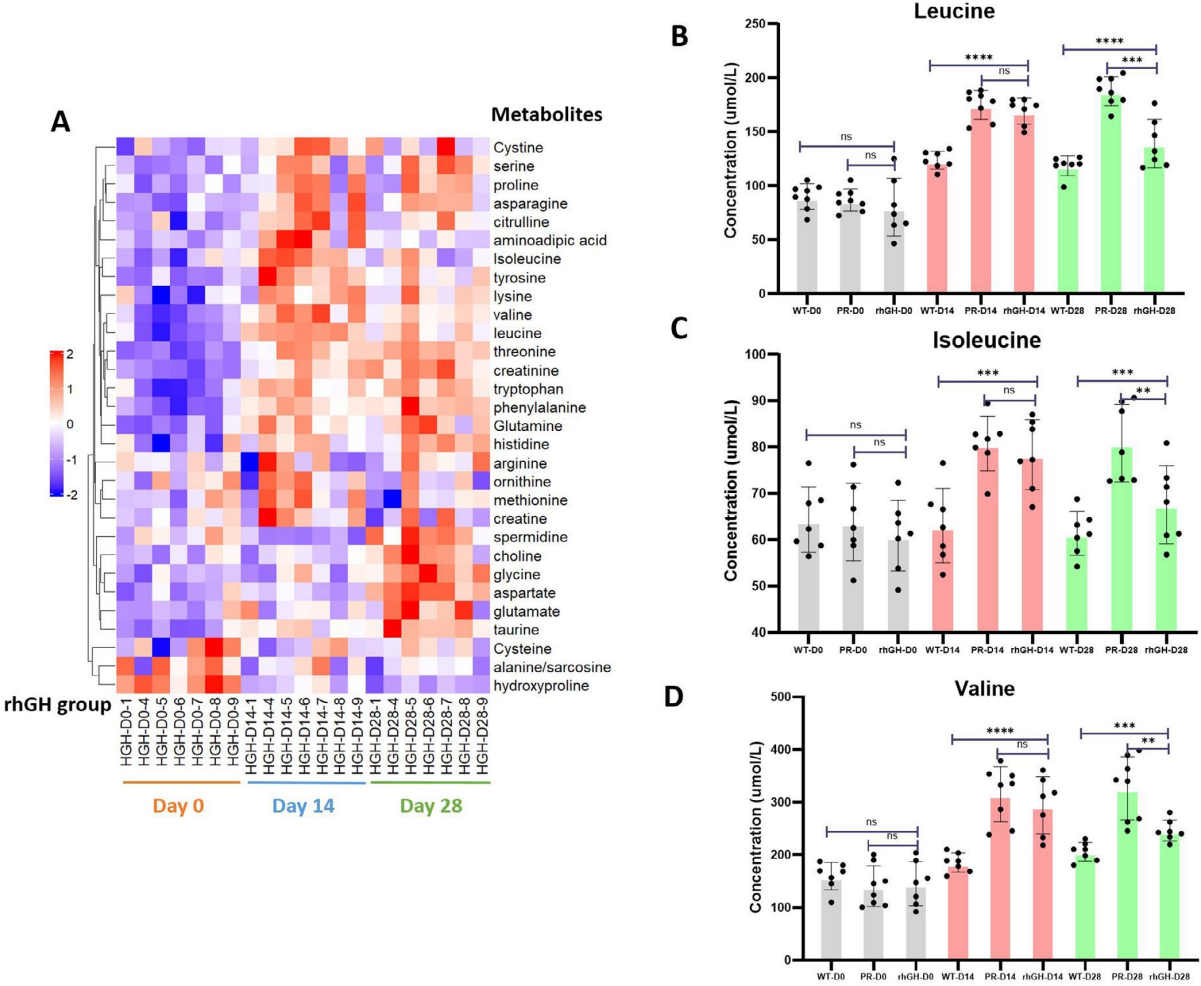
Increased BCAA concentrations in hypophysectomized rats. The heat map of amino acids or derivatives in the rhGH intervention group at different time points (A). In addition, *Z*‐score standardised metabolite concentrations were used to portray the relative quantity and changing trend of each metabolite in heatmaps by MeV software. The unit of the colour gradient was shown by *Z*‐score. The circulating concentrations of leucine (B), isoleucine (C) and valine (D) in three time points for inter‐group comparisons. ****p < 0.0001, ***p < 0.001, **p < 0.01, *p < 0.05.

Circulating amino acids were evaluated at various time points in hypophysectomized rats: prior to surgery (D0), before (D14) and after (D28) rhGH replacement. Results revealed substantial perturbation in the levels of 66.7% (20/30) amino acids or derivatives subsequent to hypophysectomy. During rhGH intervention, only four amino acids, encompassing BCAAs, such as leucine, valine, isoleucine, along with hydroxyproline, exhibited a partial normalisation of their concentrations (*p* < 0.05, *q* value < 0.2) between D14 and D28 (Figure 4A, Table 3). Specifically, the concentrations of BCAAs undergo a pronounced elevation subsequent to hypophysectomy, followed by a discernible downward trend post intervention with rhGH, as depicted in Figure 4B,D. Simultaneously, the PR group exhibited no notable variation across the Day 14 and Day 28 time points, as detailed in Figure 4E–G.

**TABLE 3:** Dynamic change of amino acids and metabolite concentrations in the serum of animal models of hypopituitarism treated with rhGH.

Metabolites	rhGH‐D0	rhGH‐D14	rhGH‐D28	rhGH‐D14 vs. rhGH‐D0		rhGH‐D28 vs. rhGH‐D14	
	Mean (μmol/L)	Mean (μmol/L)	Mean (μmol/L)	FC	*q*‐value	FC	*q*‐value
Leucine	80.10	169.10	139.12	2.11	< 0.0000	0.82	0.0625
Threonine	129.68	323.13	328.89	2.49	< 0.0000	1.08	0.4587
Creatinine	7.87	12.53	11.99	1.59	0.0001	1.14	0.2259
Phenylalanine	48.85	85.38	93.59	1.75	0.0001	1.10	0.3960
Valine	145.24	294.35	246.33	2.03	0.0003	0.84	0.1477
Hydroxyproline	62.64	37.28	29.21	0.60	0.0003	0.78	0.1360
Glutamine	857.15	1230.05	1245.34	1.44	0.0005	1.01	0.8234
Tryptophan	71.09	109.32	110.50	1.54	0.0009	1.01	0.8162
Tyrosine	55.65	97.07	81.43	1.74	0.0018	0.84	0.3049
Isoleucine	60.90	78.40	162.94	1.29	0.0018	0.86	0.1223
Taurine	119.25	176.48	216.83	1.48	0.0019	1.23	0.2067
Spermidine	3.68	1.90	5.33	0.52	0.0045	2.81	0.0006
Asparagine	54.37	82.94	78.11	1.53	0.0082	0.94	0.6513
Citrulline	136.46	181.53	162.94	1.33	0.0099	0.90	0.3784
Aminoadipic acid	1.04	1.61	1.13	1.54	0.0104	0.73	0.1256
Lysine	190.69	298.92	276.36	1.57	0.0134	0.92	0.5806
Cystine	20.62	28.07	25.66	1.36	0.0135	0.91	0.5806
Proline	189.00	258.65	29.21	1.37	0.0142	0.94	0.6513
Serine	288.01	363.03	382.48	1.26	0.0206	1.05	0.6513
Histidine	72.42	104.24	116.20	1.44	0.0391	1.11	0.3960

*Note:* rhGH‐0, blood collected before pituitary resection; rhGH‐14, blood collected 2 weeks after the operation; rhGH‐28, blood collected after 2 weeks of rhGH intervention. Mean refers to peak intensity (area).

Abbreviation: SEM, standard error of mean.

### 3.6 Regulation of BCAA Degradation and Ubiquitin‐Dependent Proteolysis in Hypophysectomized Rats

**FIGURE 5:**
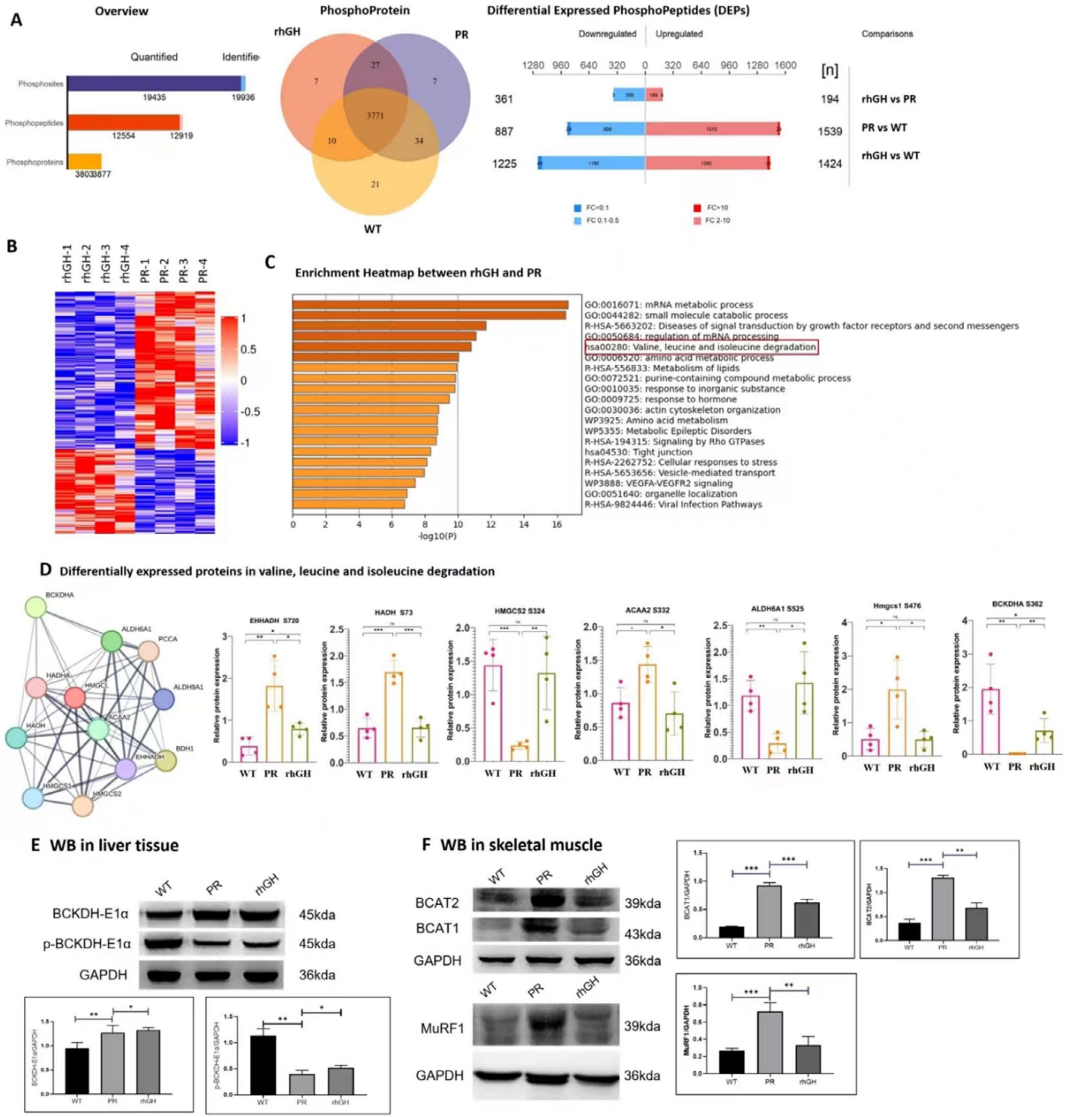
Regulation of BCAA degradation and ubiquitin‐dependent proteolysis in hypophysectomized rats. Overview and number of differentially expressed phosphoproteins (DEPPs) among WT, PR and rhGH groups (A); Heat map of DEPPs between the rhGH and PR groups (B). The unit of the colour gradient was shown by *Z*‐score. The gene set enrichment of DEPPs by KEGG between the rhGH and PR groups (C). Differences in the key enzymes related to BCAA degradation in liver tissues (D), including western blotting of BCKDHA and p‐BCKDHA (E); Western blotting of BCAT1, BCAT2 and MuRF1 in skeletal muscle among the different groups (F). ****p < 0.0001, ***p < 0.001, **p < 0.01, *p < 0.05.

To elucidate the mechanism underlying elevation of circulating BCAAs in hypopituitarism, a comprehensive 4D label‐free quantitative phosphoproteomics analysis was performed on liver tissues from WT, PR and rhGH groups. A total of 3771 phosphoproteins were evaluated across the three cohorts. A total of 194 upregulated and 361 downregulated differentially expressed phosphoproteins (DEPPs) were identified in comparison between rhGH and the PR group (Figure 5A,B). The KEGG pathways analysis revealed DEPPs between rhGH and the PR group involved in ‘mRNA metabolic process,’ ‘diseases of signal transduction by growth factor receptors and second messengers,’ and ‘valine, leucine, and isoleucine degradation’ (Figure 5C).

In the context of hypophysectomized rats, a total of 12 proteins associated with ‘valine, leucine, and isoleucine degradation pathway’ exhibited significantly perturbed phosphorylation levels. These included BCKDHA‐S362, ALDH9A1‐T28, EHHADH‐S720, HADHA‐T392, HADH‐S73, HMGCL‐S22, HMGCS1‐S476, HMGCS2‐S324, ALDH6A1‐S525, PCCA‐Y152, ACAA2‐S332 and BDH1‐S25 (Figure 5D). No significant differences were observed for the total levels of these proteins, suggesting that the effects of GH on BCAA degradation in the liver primarily occurred at the phosphorylation level rather than the protein expression level (Table S2). Among these proteins, the phosphorylation states of EHHADH‐S720, HADH‐S73, ACAA2‐S332, HMGCS1‐S476, BCKDHA‐S362 and ALDH6A1‐S525 were reversed following rhGH intervention. Specifically, the phosphorylation of BCKDHA at the S362 sites was reduced to 0.07‐fold (Figure 5D), yet it saw a substantial restoration subsequent to GH intervention. The findings from the western blotting corroborated these observations. (Figure 5E).

Given that BCAAs degradation pathway initiated in the skeletal muscle in a manner dependent on BCAT expression, we noted a significant upregulation of BCAT1 and BCAT2 in the tibialis anterior muscle of hypophysectomized rats (Figure 5F). MuRF1, an ubiquitin ligase in the ubiquitin‐proteasome‐mediated protein degradation, underwent a significant increase post‐hypophysectomy and was significantly restored subsequent to the administration of rhGH.

## 4 Discussion

In human cohorts, BCAA and related metabolites are now widely recognised as among the strongest biomarkers of obesity, IR, T2D and cardiovascular diseases [11]. Within the hypopituitarism cohort, a significant elevation in BCAA levels was observed in patients with hypopituitarism compared to healthy controls. Furthermore, there was a positive correlation identified between BCAA concentrations and both triglycerides and IR levels. However, no definitive conclusions can be drawn regarding the potential causal role of BCAAs in disease pathogenesis, based on current correlative metabolic data alone.

IR is characterised by reduced sensitivity or responsiveness to the metabolic actions of insulin, encompassing defects in glucose uptake and oxidation, diminished glycogen synthesis and impaired suppression of lipid oxidation [20]. Felig et al. proposed that the elevated levels of BCAAs and aromatic amino acids observed in individuals with obesity may be a consequence, rather than a cause, of IR [21]. However some evidence suggests that BCAAs may independently contribute to IR. Metabolomic studies have indicated that elevated BCAA levels in individuals with normal fasting glycemia are associated with an increased risk of IR and diabetes [11]. Recent human genetic studies investigating variants affecting insulin sensitivity and lipid traits in relation to BCAA levels [22, 23] have proposed a unifying model. According to this model, increases in BCAA observed in pre‐diabetic individuals with obesity may primarily result from IR. However, once elevated, BCAAs could potentially play a causal role in the progression from prediabetes to full‐blown diabetes [24]. It is plausible that elevated BCAAs may be a consequence of IR, yet they might subsequently exert an influence on lipid and glucose metabolism within the context of hypopituitarism. The precise role of BCAAs in hypopituitarism, whether they are mere consequences, causative factors or mere biomarkers of impaired insulin response, remains to be elucidated.

The regulation of circulating BCAAs involves various factors, such as dietary consumption, protein synthesis or oxidation, and the rate of proteolysis and release of free amino acids, while de novo biosynthesis is not observed in human tissues [25]. To mitigate the influence of diet‐derived amino acids, known to cause a notable increase in BCAA levels post‐ingestion of animal protein‐rich meals, we assessed amino acid concentrations under fasting conditions. In the hypophysectomized rat model, which is characterised by a severe loss of appetite and a lack of obvious weight gain during the experiment, the elevation of fasting BCAAs is unlikely to be attributed to an excess of diet‐derived amino acids.

On the contrary, the notable reduction in the weight or volume of skeletal muscles, as well as the increased density of muscle fibres, indicates the occurrence of muscle atrophy. The mice with GH deficiency showed smaller muscle fibres and normal muscle function according to Mavalli's research [26]. Due to the remarkable degree of intragroup variability within the PR and rhGH groups, there is no significant difference observed in CSA. The compaction of muscle fibres may be related to muscle atrophy, as the reduced interstitial space can impair the nutritional supply to the muscle fibres, potentially triggering their atrophy. Several studies have noted that a more compact arrangement may be associated with a shift in muscle fibre type towards the more densely packed slow‐twitch fibres [27]. Additionally, the substantial increase in the levels of most amino acids and derivatives in both patients with hypopituitarism and the hypophysectomized rat model corroborates the state of enhanced proteolysis. This is further supported by the heightened expression of MuRF1, a key enzyme in the ubiquitin‐proteasome‐mediated protein degradation pathway. Collectively, the increased concentrations of BCAAs may be indicative of elevated proteolysis during the post‐absorptive state. Holeček et al. have proposed that skeletal muscle plays a dominant role in BCAA catabolism and that activated proteolysis and IR can contribute to the elevation of BCAA levels [28]. The ubiquitin‐proteasome system (UPS) is implicated in the degradation of major skeletal muscle proteins and plays a significant role in muscle wasting. MAFbx and MuRF1, as ubiquitin‐protein ligases, are crucial components of the UPS system [29]. Although there is no direct evidence to suggest that GH regulates the expression of MAFbx and MuRF1, one study has shown that Ghrelin, which restores plasma GH levels in burned rats, can decrease proteolysis by modulating MuRF1 and MAFbx [30]. Furthermore, a significant reduction in circulating BCAA levels was observed during fasting with GH replacement therapy, attributed to diminished proteolysis [31, 32]. In the current study, the expression of MuRF1 was significantly augmented following hypophysectomy, a yet it could be mitigated by rhGH intervention, implying its role as a marker of muscle proteolysis regulated by GH.

The application of 4D label‐free quantitative phosphoproteomics has revealed the KEGG pathways that are significantly enriched and distinguish the rhGH group from the PR group. Notably, these include pathways involved in ‘mRNA metabolism,’ ‘diseases of signal transduction by growth factor receptors and second messengers,’ and ‘valine, leucine, and isoleucine degradation.’ The inclusion of ‘diseases of signal transduction by growth factor receptors and second messengers’ is rational, given the two‐week rhGH intervention on hypophysectomized rats. Among the 12 proteins exhibiting dysregulated phosphorylation levels in the ‘valine, leucine, and isoleucine degradation pathway,’ the rate‐limiting enzyme BCKDHA in the liver's BCAA degradation pathway showed significantly dephosphorylation. BCKDHA is the α‐subunit of the E1 component of the BCKDH complex, which is responsible for catalysing the second step critical and irreversible reaction in BCAA degradation. The activity of BCKDHA is regulated by BCKDK kinase and PPM1K phosphatase. BCKDK kinase inhibits the activity of BCKDHA through phosphorylation, while PPM1K activates the BCKDH complex by dephosphorylating BCKDHA [16, 33]. Although the phosphorylation site identified in our proteomic analysis was BCKDHA‐S362, we employed the canonical BCKDHA‐Ser293 antibody during the WB validation and obtained a consistent trend. Therefore, the dephosphorylation state of BCKDHA in PR group is an indication of an enhanced BCAA degradation.

BCATs are responsible for the first step in the catabolism of BCAAs, catalysing the reversible transamination reaction between BCAAs and their corresponding α‐keto acids (BCKAs). A decline in BCATs expression may lead to a reduction in BCAA catabolism, thereby affecting the concentration of BCAAs in the serum. Evidence including the deficiency of BCAT2 has been demonstrated to reduce circulating BCAA levels by 30%–50% [17]. BCAT2 enhances BCAA uptake to sustain BCAA catabolism and mitochondrial respiration in the development of pancreatic ductal adenocarcinoma [34]. We found the expression of BCATs increased in the PR group, which may indicate an enhanced BCAA degradation, like BCKDHA dephosphorylation. Collectively, these findings suggest that the BCAA degradation pathway is activated in the context of GH deficiency, a state that appears amenable to correction through rhGH intervention. These results were unexpected, given that as IR‐related diseases, such as obesity, typically entail a broad transcriptional repression of the BCAA degradation pathway. The underlying mechanism behind this phenomenon remains unexplored, especially considering the scarcity of research investigating the interaction between GH and BCAA oxidation. Further investigations are warranted to unravel the intricate details of the relationship between GH and BCAA metabolism in GHD.

Despite the promising findings, the present study has notable limitations. Firstly, BCAA metabolism has been shown to be sex‐dependent in previous studies [35, 36], which may limit the generalizability of our findings since only male patients and animal models were included in this study. Secondly, we did not collect data on patients' daily protein intake and activity levels, which can also have an impact on their BCAA metabolism. Thirdly, hypophysectomized rats were chosen as the animal model for hypopituitarism, rather than employing GH receptor knockout transgenic rats. The choice of hypophysectomized rats enabled the demonstration of GH's compensatory effect; however, it also hindered the exploration of potential effects from other pituitary hormones, as no hormone replacement therapies were provided to the rats. Fourthly, the analysis of muscle fibre type and functional study has not been conducted. However, such an analysis is only applicable to fresh samples. This limitation is primarily due to methodological issues, as such analyses are only applicable to fresh samples. Our samples have already been fixed and embedded in paraffin, thus missing the optimal time for detection. To compensate for this missing content, we assessed the cellular arrangement density, as there are certain differences in the arrangement of different fibre types. Generally, slow‐twitch fibres (Type I) have a higher density and more compact arrangement compared to fast‐twitch fibres (Type II). Future work will focus on addressing these methodological limitations by conducting experiments on fresh samples to analyse muscle fibre types and their functional characteristics. Additionally, without data on other comorbidities that patients may have, which can also alter BCAA metabolism. Furthermore, the patients enrolled in our study did not undergo rhGH therapy in adulthood due to economic constraints or concerns related to tumour recurrence, cancer and diabetes risks. Consequently, we were unable to observe the impact of GH intervention on BCAA levels in patients with hypopituitarism.

**FIGURE 6:**
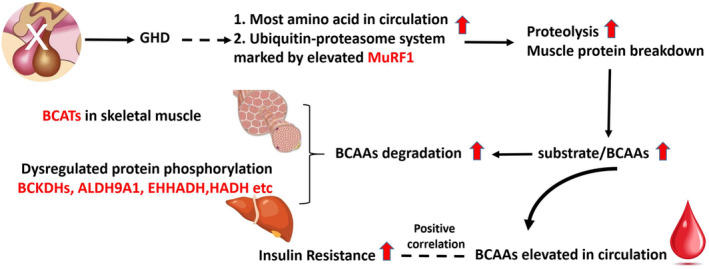
The model of BCAAs catabolism in hypopituitarism. The removal of the pituitary led to growth hormone deficiency. GH deficiency promotes proteolysis in skeletal muscle through the MURF1‐mediated ubiquitin‐proteasome system, which leads to higher levels of BCAAs in the bloodstream. The BCAAs degradation was activated through expressing BCATs in skeletal muscle and dephosphorylated BCKDHs in liver tissues, due to increased substrate concentration from proteolysis. The elevated BCAAs have a positive correlation with insulin resistance. However, it remains unclear whether the elevation of BCAAs is a consequence or a cause of insulin resistance.

In conclusion, our study establishes a clear association between elevated circulating BCAAs and IR in hypopituitarism. The activation of the BCAA degradation pathway in the state of GHD suggests a complex interplay between GH and BCAA metabolism. The observed increase in fasting BCAAs likely stems from augmented proteolysis and IR, contributing to elevated BCAA levels in the bloodstream that exceed their degradation and utilisation (Figure 6). While these findings provide valuable insights into the impact of GH on BCAA catabolism, the study's limitations underscore the necessity for additional investigations to refine our comprehension and potentially contribute to the creation of innovative therapeutic strategies.

## Author Contributions

**Yuwen Zhang:** conceptualization (equal), formal analysis (equal), funding acquisition (equal), investigation (equal), resources (equal), validation (equal), writing – original draft (equal), writing – review and editing (equal). **Zhiqiu Ye:** data curation (equal), methodology (equal), resources (equal), validation (equal), writing – review and editing (equal). **Enfei Xiang:** methodology (equal), writing – review and editing (equal). **Peizhan Chen:** conceptualization (equal), funding acquisition (equal), investigation (equal), methodology (equal), project administration (equal), supervision (equal), writing – review and editing (equal). **Xuqian Fang:** conceptualization (equal), formal analysis (equal), funding acquisition (equal), investigation (equal), methodology (equal), project administration (equal), supervision (equal), writing – original draft (equal), writing – review and editing (equal).

## Conflicts of Interest

The authors declare no conflicts of interest.

## Supporting information


Data S1.

Data S2.

## Data Availability

The data and materials used or analyzed during the current study are available from the corresponding author Prof. Xuqian Fang (251720307@qq.com) on reasonable request.
