# Supplementary material for: Effect of Growth Hormone on Branched‐Chain Amino Acids Catabolism in Males With Hypopituitarism
Source: J Cell Mol Med. 2025 Mar 3;29(5):e70451. doi: 10.1111/jcmm.70451 (PMC11875759; doi:10.1111/jcmm.70451)

**Supplement Figure 1** The phenotype and weight of the muscle of thigh.


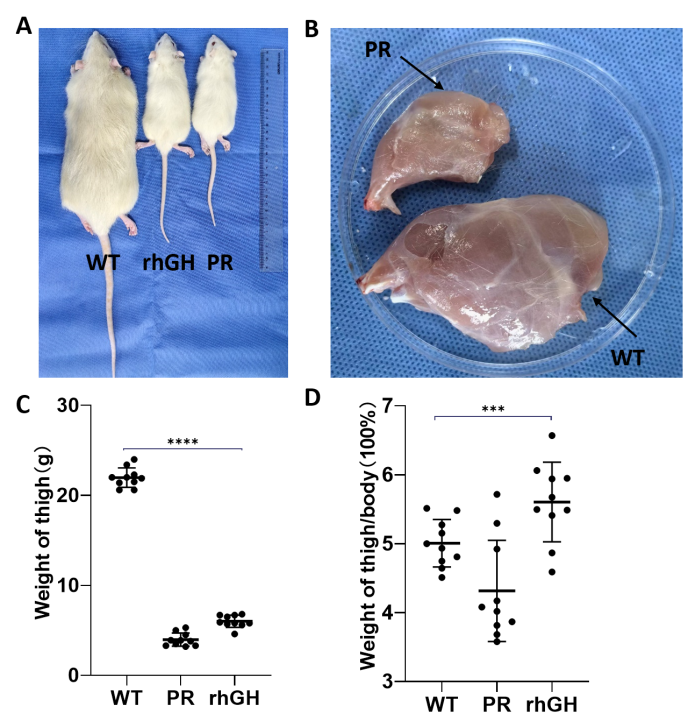


**Supplement Figure 2** The assessment of cell density in Skeletal Muscle for WT group.

**
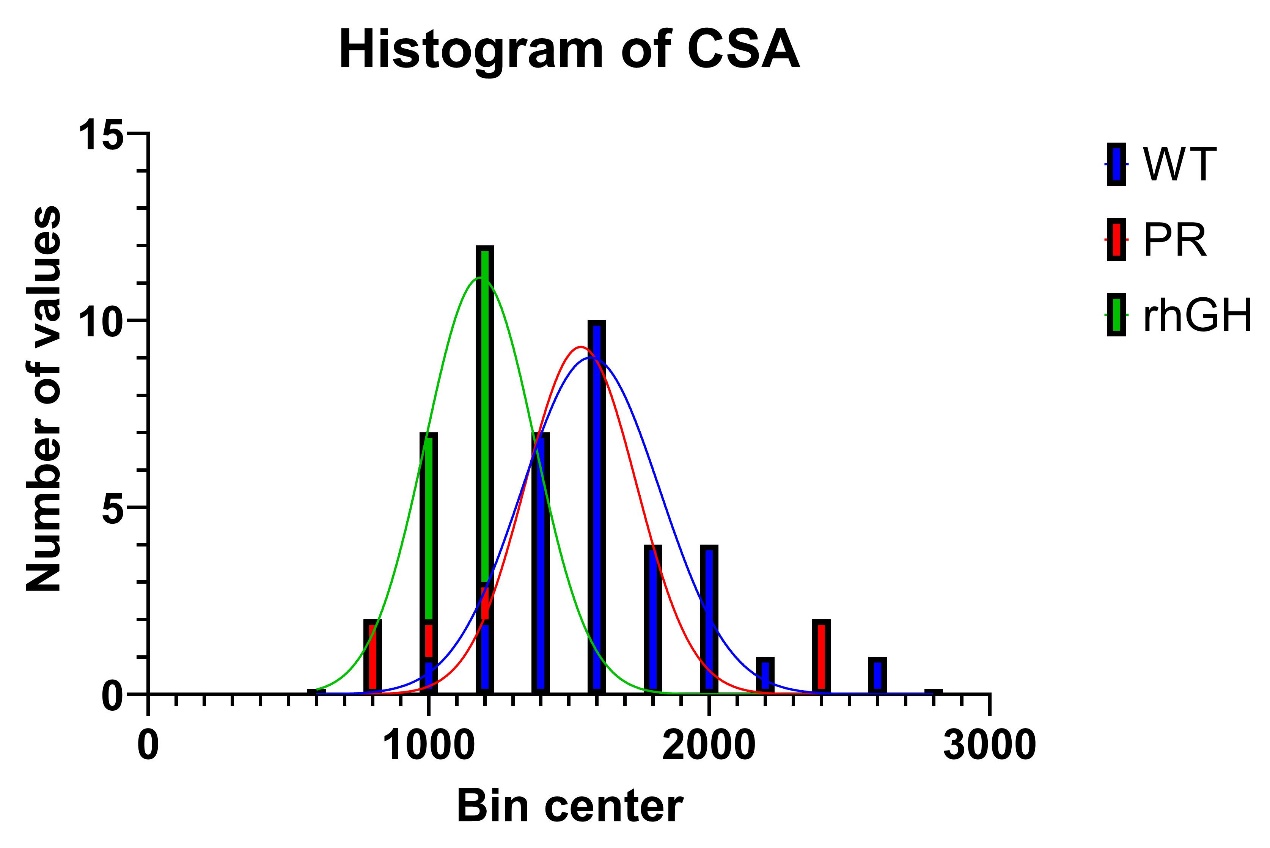
**

**Supplement Figure 3** The assessment of cell density in Skeletal Muscle for WT group.


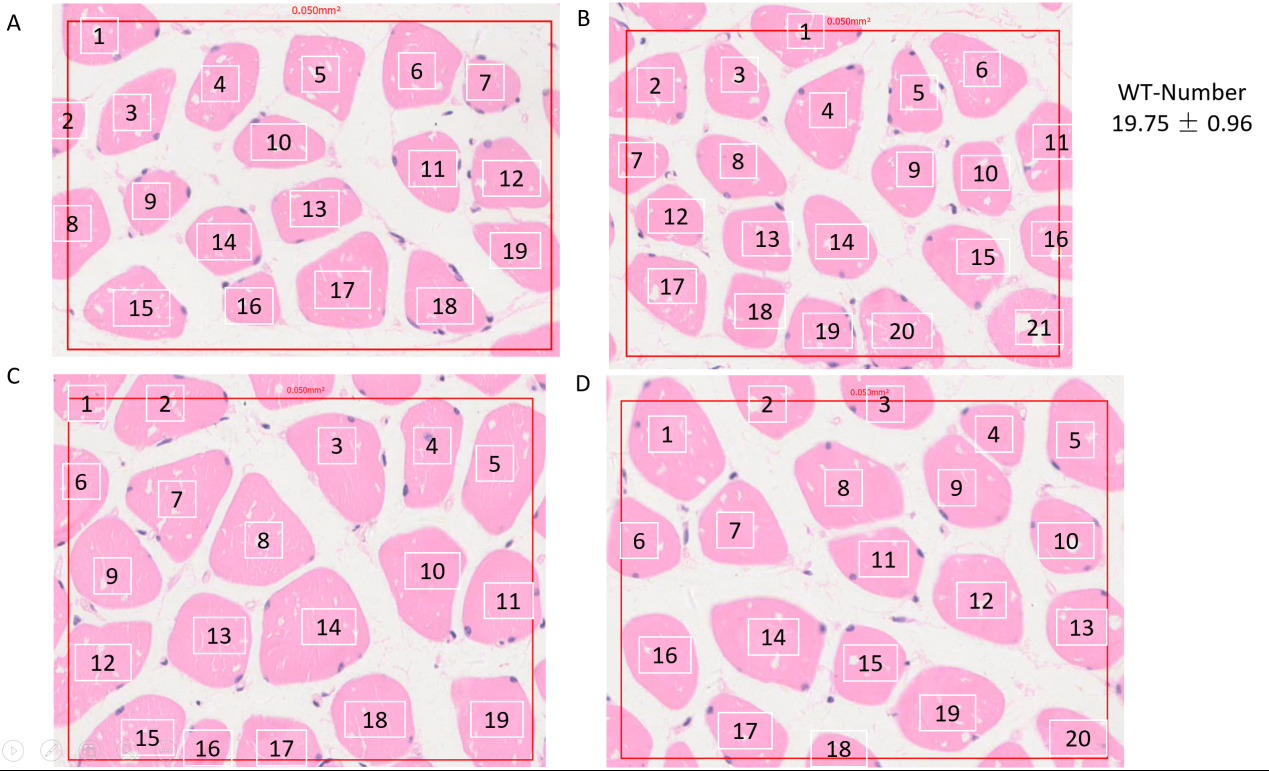


**Supplement Figure 4** The Assessment of cell density in Skeletal Muscle for PR group.


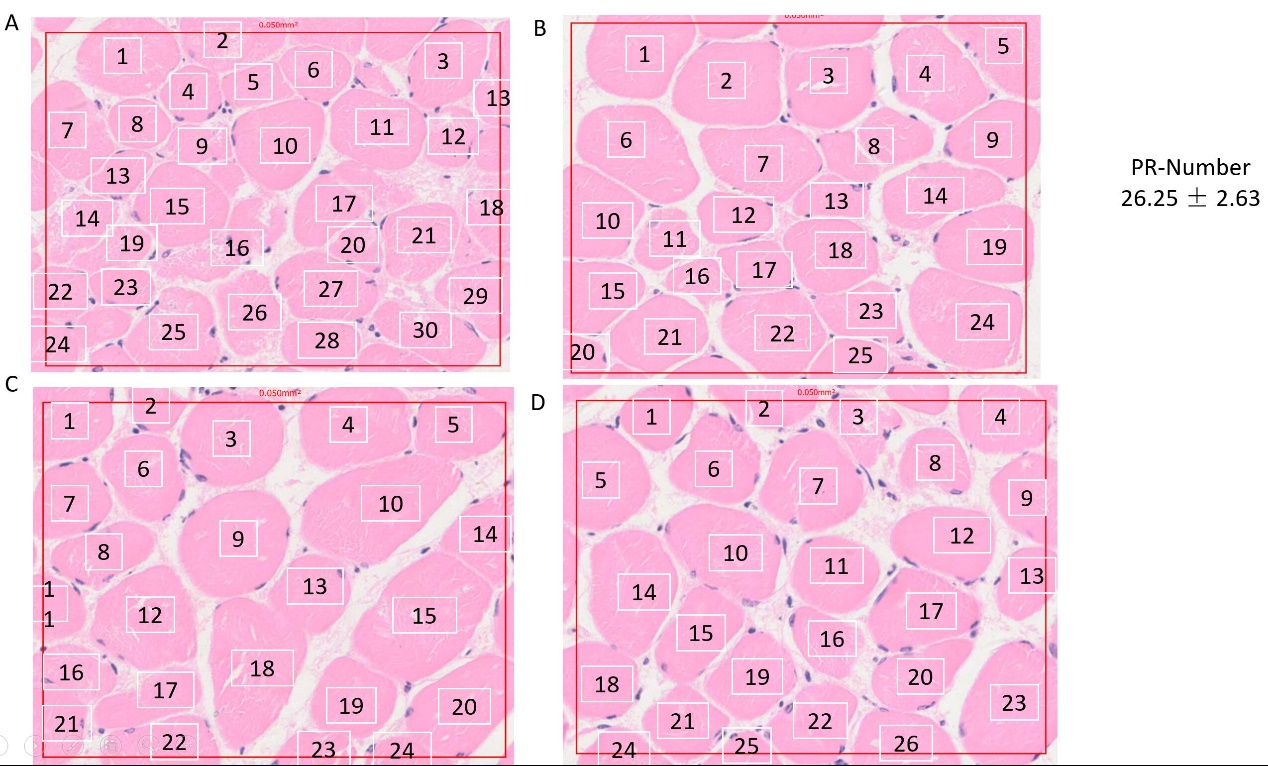


**Supplement Figure 5** The Assessment of cell density in Skeletal Muscle for rhGH group.


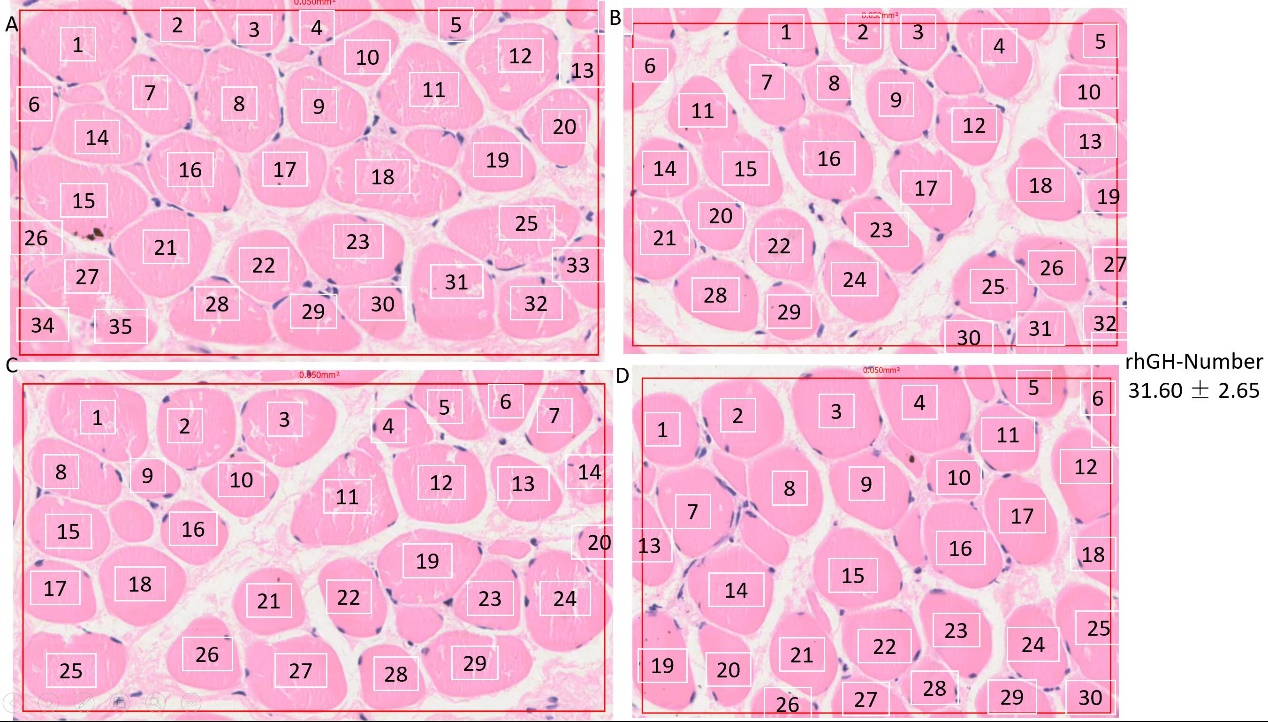

Supplement: Supplementary file 1 — Data S1. [file JCMM-29-e70451-s001.docx]
