# Supplementary material for: Effect of Growth Hormone on Branched‐Chain Amino Acids Catabolism in Males With Hypopituitarism
Source: J Cell Mol Med. 2025 Mar 3;29(5):e70451. doi: 10.1111/jcmm.70451 (PMC11875759; doi:10.1111/jcmm.70451)
Supplement: Supplementary file 2 — Data S2. [file JCMM-29-e70451-s002.docx]

**Supplement Table 1** The food intake and weight gain in animal models of hypopituitarism

|  |  | Weight（g） | | | | Daily consumed food (g) |
| --- | --- | --- | --- | --- | --- | --- |
| Groups | Rat ID | D0 | D14 | △weight | change（%） | D0--D14 |
| PR group | 1# | 77.6 | 80.8 | 3.2 | 4.1 | 5 |
|  | 2# | 83.1 | 86.5 | 3.4 | 4.1 | 4.4 |
|  | 3# | 75.6 | 78.8 | 3.2 | 4.2 | 5.5 |
|  | 4# | 80.2 | 85 | 4.8 | 6.0 | 6.3 |
|  | 5# | 78.2 | 80.4 | 2.2 | 2.8 | 5 |
|  | 6# | 78 | 79.8 | 1.8 | 2.3 | 5.8 |
|  | 7# | 81.8 | 83.6 | 1.8 | 2.2 | 3.4 |
|  | 8# | 89.1 | 93.8 | 4.7 | 5.3 | 6.3 |
|  | 9# | 88.9 | 93.2 | 4.3 | 4.8 | 6.3 |
|  | 10# | 84.5 | 87.9 | 3.4 | 4.0 | 5.4 |
| rhGH group | 1# | 92.2 | 87.1 | -5.1 | -5.5 | 5.5 |
|  | 2# | 91.4 | 93.6 | 2.2 | 2.4 | 5.8 |
|  | 3# | 98.7 | 99 | 0.3 | 0.3 | 6.5 |
|  | 4# | 89.2 | 93 | 3.8 | 4.3 | 6.7 |
|  | 5# | 89.1 | 94.6 | 5.5 | 6.2 | 6.1 |
|  | 6# | 89.5 | 91.3 | 1.8 | 2.0 | 6 |
|  | 7# | 89.5 | 94.8 | 5.3 | 5.9 | 6 |
|  | 8# | 95.2 | 97.4 | 2.2 | 2.3 | 5.7 |
|  | 9# | 86.2 | 90.7 | 4.5 | 5.2 | 5.9 |
|  | 10# | 92.5 | 94 | 1.5 | 1.6 | 6 |
| WT group | 1# | 96.2 | 149 | 52.8 | 54.9 | 19.6 |
|  | 2# | 85.1 | 141.4 | 56.3 | 66.2 | 20.3 |
|  | 3# | 75.1 | 129 | 53.9 | 71.8 | 22.9 |
|  | 4# | 85.8 | 150 | 64.2 | 74.8 | 24.3 |
|  | 5# | 74.2 | 125 | 50.8 | 68.5 | 25.4 |
|  | 6# | 83.1 | 146.3 | 63.2 | 76.1 | 21.8 |
|  | 7# | 81.4 | 143.9 | 62.5 | 76.8 | 22.5 |
|  | 8# | 77.8 | 132.1 | 54.3 | 69.8 | 22.6 |
|  | 9# | 71.4 | 116.1 | 44.7 | 62.6 | 22.9 |
|  | 10# | 89.3 | 149.3 | 60 | 67.2 | 23.4 |

For weight change %, PR vs. rhGH *p* = 0.918；PR vs. WT *p* <0.0001；rhGH vs. WT *p* <0.0001；analyzed by one way ANOVA.

For Daily consumed food, PR vs. rhGH *p* = 0.143；PR vs. WT *p* <0.0001；rhGH vs. WT *p* <0.0001；analyzed by two way ANOVA.

**Supplement Table 2** The protein expression level of key enzymes in BCAAs degradation.

| Gene  Name | Mol  Weight | Average PR  (intensity) | Average PR-rhGH  (intensity) | PR-hGH/PR | t test  *p* value |
| --- | --- | --- | --- | --- | --- |
| Bckdha | 52.87 | 18656250 | 18274000 | 0.98 | 0.81 |
| Aldh9a1 | 54.05 | 79273750 | 79417250 | 1.00 | 0.98 |
| Ehhadh | 78.66 | 148145000 | 160090000 | 1.08 | 0.52 |
| Hadha | 82.66 | 54401500 | 55565000 | 1.02 | 0.67 |
| Hadh | 34.45 | 23806750 | 24786000 | 1.04 | 0.35 |
| Hmgcl | 34.19 | 6153575 | 6261500 | 1.02 | 0.81 |
| Hmgcs1 | 57.43 | 6713575 | 5096975 | 0.76 | 0.16 |
| Hmgcs2 | 56.89 | 172445000 | 155545000 | 0.90 | 0.18 |
| Aldh6a1 | 57.75 | 160525000 | 161945000 | 1.01 | 0.90 |
| Pcca | 79.81 | 18091750 | 17510750 | 0.97 | 0.30 |
| Acaa2 | 41.75 | 205692500 | 160725000 | 0.78 | 0.05 |
| Bdh1 | 38.33 | 114265000 | 113600000 | 0.99 | 0.90 |

**Supplement Table 3** The Calculation of Muscle Cross-sectional Area

| Groups | L | W | A | Groups | L | W | A | Groups | L | W | A | *P* |
| --- | --- | --- | --- | --- | --- | --- | --- | --- | --- | --- | --- | --- |
| WT-1 | 49.5 | 35.0 | 1360.0 | PR-1 | 57.8 | 51.5 | 2334.7 | GH-1 | 51.6 | 41.0 | 1660.7 | ANOVA |
| WT-2 | 51.3 | 34.0 | 1369.2 | PR-2 | 42.0 | 31.0 | 1022.1 | GH-2 | 45.7 | 33.7 | 1209.0 | 0.0002 |
| WT-3 | 66.0 | 37.5 | 1942.9 | PR-3 | 55.0 | 37.5 | 1619.1 | GH-3 | 54.5 | 32.4 | 1386.2 | WT vs. PR |
| WT-4 | 58.0 | 39.5 | 1798.4 | PR-4 | 37.0 | 28.0 | 813.3 | GH-4 | 48.2 | 31.2 | 1180.5 | 0.63 |
| WT-5 | 55.6 | 33.0 | 1440.3 | PR-5 | 49.0 | 39.0 | 1500.1 | GH-5 | 37.2 | 31.4 | 916.9 |  |
| WT-6 | 51.3 | 35.0 | 1409.5 | PR-6 | 33.0 | 28.0 | 725.3 | GH-6 | 47.6 | 41.0 | 1532.0 | WT vs. rhGH |
| WT-7 | 55.5 | 36.0 | 1568.4 | PR-7 | 55.5 | 42.0 | 1829.8 | GH-7 | 51.2 | 35.0 | 1406.7 | 0.0003 |
| WT-8 | 56.0 | 34.5 | 1516.6 | PR-8 | 53.0 | 39.0 | 1622.6 | GH-8 | 36.5 | 28.4 | 813.7 |  |
| WT-9 | 53.5 | 35.0 | 1469.9 | PR-9 | 54.0 | 39.0 | 1653.2 | GH-9 | 45.7 | 28.6 | 1026.0 | PR vs. rhGH |
| WT-10 | 53.5 | 37.0 | 1553.9 | PR-10 | 45.0 | 35.0 | 1236.4 | GH-10 | 48.5 | 35.2 | 1340.2 | 0.0060 |
| WT-11 | 58.0 | 39.0 | 1775.7 | PR-11 | 57.0 | 30.7 | 1373.7 | GH-11 | 43.0 | 34.0 | 1147.7 |  |
| WT-12 | 51.9 | 31.3 | 1274.9 | PR-12 | 56.0 | 36.8 | 1617.7 | GH-12 | 42.7 | 32.5 | 1089.4 |  |
| WT-13 | 67.6 | 33.3 | 1765.0 | PR-13 | 51.3 | 36.8 | 1478.5 | GH-13 | 43.8 | 35.0 | 1203.4 |  |
| WT-14 | 53.3 | 39.6 | 1655.3 | PR-14 | 53.8 | 36.6 | 1544.3 | GH-14 | 45.8 | 37.2 | 1336.0 |  |
| WT-15 | 51.6 | 48.9 | 1980.8 | PR-15 | 53.5 | 34.0 | 1427.9 | GH-15 | 43.2 | 31.3 | 1061.4 |  |
| WT-16 | 63.0 | 46.0 | 2274.9 | PR-16 | 47.5 | 42.3 | 1575.4 | GH-16 | 63.2 | 42.5 | 2108.5 |  |
| WT-17 | 66.0 | 37.9 | 1963.6 | PR-17 | 59.0 | 41.0 | 1898.9 | GH-17 | 44.0 | 35.5 | 1226.2 |  |
| WT-18 | 51.9 | 37.6 | 1531.9 | PR-18 | 60.0 | 32.0 | 1507.2 | GH-18 | 57.0 | 37.2 | 1664.5 |  |
| WT-19 | 59.0 | 39.6 | 1834.1 | PR-19 | 44.0 | 35.0 | 1208.9 | GH-19 | 43.6 | 36.8 | 1259.5 |  |
| WT-20 | 47.9 | 41.3 | 1552.9 | PR-20 | 70.0 | 44.0 | 2417.8 | GH-20 | 43.0 | 30.3 | 1022.8 |  |
| WT-21 | 61.9 | 52.0 | 2526.8 | PR-21 | 65.0 | 52.0 | 2653.3 | GH-21 | 45.5 | 36.0 | 1285.8 |  |
| WT-22 | 66.2 | 37.3 | 1937.7 | PR-22 | 45.0 | 38.0 | 1342.4 | GH-22 | 46.6 | 32.7 | 1196.2 |  |
| WT-23 | 54.3 | 35.3 | 1502.4 | PR-23 | 51.0 | 41.0 | 1641.4 | GH-23 | 46.0 | 31.5 | 1137.5 |  |
| WT-24 | 44.3 | 41.3 | 1433.6 | PR-24 | 38.0 | 34.0 | 1014.2 | GH-24 | 46.0 | 33.0 | 1191.6 |  |
| WT-25 | 39.6 | 35.6 | 1107.0 | PR-25 | 49.0 | 41.0 | 1577.1 | GH-25 | 42.0 | 35.0 | 1154.0 |  |
| WT-26 | 55.91 | 32.9 | 1444.0 | PR-26 | 55.0 | 34.0 | 1468.0 | GH-26 | 51.0 | 39.0 | 1561.4 |  |
| WT-27 | 51.3 | 41.9 | 1686.9 | PR-27 | 46.0 | 34.0 | 1227.7 | GH-27 | 41.6 | 30.3 | 989.5 |  |
| WT-28 | 60.2 | 35.3 | 1669.3 | PR-28 | 72.0 | 37.0 | 2091.2 | GH-28 | 44.0 | 31.0 | 1070.7 |  |
| WT-29 | 56.9 | 35.3 | 1576.7 | PR-29 | 47.0 | 36.0 | 1328.2 | GH-29 | 43.0 | 35.0 | 1181.4 |  |
| WT-30 | 39.6 | 31.6 | 982.9 | PR-30 | 57.0 | 38.0 | 1700.3 | GH-30 | 54.0 | 38.0 | 1610.8 |  |
